# Draft Genome Sequence of *Bacillus safensis* JPL-MERTA-8-2, Isolated from a Mars-Bound Spacecraft

**DOI:** 10.1128/genomeA.01360-15

**Published:** 2015-11-19

**Authors:** David A. Coil, James N. Benardini, Jonathan A. Eisen

**Affiliations:** a University of California Davis Genome Center, Davis, California, USA; b Jet Propulsion Laboratory (JPL), Pasadena, California, USA; c Department of Evolution and Ecology, University of California Davis, Davis, California, USA; d Department of Medical Microbiology and Immunology, University of California Davis, Davis, California, USA

## Abstract

Here, we present the draft genome of *Bacillus safensis* JPL-MERTA-8-2, a strain found in a spacecraft assembly cleanroom before launch of the Mars Exploration Rovers. The assembly contains 3,671,133 bp in 14 contigs.

## GENOME ANNOUNCEMENT

As part of standard Planetary Protection protocols at JPL-NASA, all Mars-bound spacecraft are routinely sampled to monitor contamination within the spacecraft assembly cleanrooms. The swabs and wipes are cultured and banked for further study. This particular isolate, *Bacillus safensis* JPL-MERTA-8-2, was collected in 2004 from soft goods (e.g., lander petal fabric) of the Exploration Rovers before launch. This isolate was recently sent to the International Space Station as part of a nationwide citizen science project, Project MERCCURI (http://www.spacemicrobes.org).

All the bacterial strains associated with this project had their genomes sequenced at the University of California Davis, as described below. Genomic DNA was extracted using a Wizard Genomic DNA purification kit (Promega) from fresh overnight cultures. Illumina paired-end libraries were made following the manufacturer’s protocol using a Nextera DNA library preparation kit (Illumina).

A total of 11,927,30 paired-end reads were generated on an Illumina MiSeq, at a read length of 300 bp. Quality trimming and error correction resulted in 11,655,85 high-quality reads. All sequence processing and assembly was performed using the A5 assembly pipeline (1) (version A5-miseq 20140604). The assembly produced 14 contigs (*N*_50_ = 1,906,962), totaling 3,671,133 bp, with a GC content of 42% and an estimated overall coverage of 70×. Completeness of the genome was assessed using Phylosift (2), which searches for 37 highly conserved, single-copy marker genes (3), all of which were found in this assembly.

Automated annotation was performed using the RAST server (4). *Bacillus safensis* JPL-MERTA-8-2 contains 3,818 predicted protein coding sequences and 95 predicted noncoding RNAs. An almost full-length (1,139 bp) 16S sequence was obtained from this annotation and was used to confirm the identity of the isolate.

### Nucleotide sequence accession numbers.

This whole-genome shotgun project has been deposited at DDBJ/EMBL/GenBank under the accession number LATH00000000. The version described in this paper is the first version, LATH01000000.
